# Association of leisure and occupational physical activities and health-related quality of life: Tehran Lipid and Gluycose Study

**DOI:** 10.1186/s12955-020-1272-0

**Published:** 2020-01-20

**Authors:** Sara Jalali-Farahani, Parisa Amiri, Kiana Torshizi, Leila Cheraghi, Masoume AvatefFazeli, Fereidoun Azizi

**Affiliations:** 1 Research Center for Social Determinants of Health, Research Institute for Endocrine Sciences, Shahid Beheshti University of Medical Sciences, P.O.Box: 19395-4763, Tehran, IR Iran; 2 Students’ Research Committee, Shahid Beheshti University of Medical Sciences, Tehran, Iran; 3 Dalla Lana School of Public Health, University of Toronto, Toronto, Canada; 4 Biostatistics Department, Research Institute for Endocrine Sciences, Shahid Beheshti University of Medical Sciences, Tehran, Iran; 5 Endocrine Research Center, Research Institute for Endocrine Sciences, Shahid Beheshti University of Medical Sciences, Tehran, Iran

**Keywords:** Health-related quality of life, Physical activity, Adults, Iran

## Abstract

**Background:**

Little is known about the association between different levels of physical activity (PA) and health-related quality of life (HRQoL) in the general Iranian population across sex groups. The current study aims to investigate the association between PA and HRQoL across sex groups, various types of physical activity (leisure time and occupational) and different dimensions of HRQoL in a large population of Tehranian adults.

**Methods:**

This cross-sectional study was conducted using data from the Tehran Lipid and Glucose Study (TLGS). Data was collected from 7800 adults on their PA habits and HRQoL. Information on PA and HRQoL were assessed using the Modifiable Activity Questionnaire (MAQ) and Short-Form 12-Item Health Survey version 2 (SF-12v2), respectively. Poor HRQoL was defined as the first quartile of HRQoL scores and logistic regression analysis was used to assess the association between physical activity levels and poor HRQoL.

**Results:**

The mean age of participants was 46.4 ± 14.9 years and 45.6% of them were male. Levels of PA were significantly associated with most subscales of HRQoL in both men (*p* < 0.05) and women (*p* < 0.01). In both sexes, leisure time PA was significantly correlated to all subscales of HRQoL (*p* < 0.05) except for bodily pain in both sexes and for social functioning and role emotional in men. In adjusted models, men with both moderate (OR: 1.55, 95%CI: 1.18–2.04; *p* = 0.002) and low (OR: 1.46, 95%CI: 1.11–1.91; *p* = 0.007) levels of PA had a significantly higher chance of reporting poor mental component summary (MCS) compared to their counterparts with high levels of PA. Furthermore, women with low levels of PA had a significantly higher chance of reporting poor physical component summary (PCS) (OR: 2.39, 95% CI: 1.63–3.49; *p* < 0.001) compared to those with high levels of PA.

**Conclusion:**

The findings show an association between PA and both domains of HRQoL in men and mostly the physical domain in women, suggesting a sex-specific pattern for this association, which could be considered to motivate participation in PA programs in future health promotion interventions.

## Introduction

Physical activity (PA) defined as any bodily movement that is accompanied by energy expenditure is a significant lifestyle behavior. PA is found to be associated with lower risk of various chronic diseases including heart problems, high blood pressure, breathing problems, allergies, type 2 diabetes, and cancers [1]. In addition to objective health outcomes, PA has been found to be associated with different aspects of self-reported subjective outcomes including happiness, life satisfaction, positive affect and health-related quality of life (HRQoL) [2–5].

HRQoL is a multidimensional concept that refers to aspects of quality of life related to an individual’s perception of their physical, mental and social domains of health. This important health outcome is widely considered in planning, implementation and evaluation of health programs. Identifying factors associated with HRQoL can help to inform health policymakers with regards to resource allocation decisions. Existing evidence indicates that socio-demographic variables, chronic illness, environmental and behavioral factors are associated with HRQoL [5–9]. Among all determinants of HRQoL, modifiable factors such as smoking habits, dietary and PA patterns play a critical role in the design of HRQoL interventions. The association between PA and HRQoL in various populations has been well documented [5, 10–12]. Existing cross-sectional and longitudinal studies have found positive associations between leisure time PA and HRQoL [13–16]. However, evidence regarding the association between occupational PA and HRQoL are limited and inconsistent as some findings showed a positive association between occupational PA and subdomains of HRQoL [13, 15] while others indicated a negative associations, specifically in males [15, 17].

While there is a relatively rich literature regarding association between PA and HRQoL in other countries, this association has not been adequately addressed in the general Iranian population. Existing relevant evidence in Iran has focused on specific groups of women including those who were exercising in gymnasiums, middle aged and elderly groups [18–20]. Considering the findings that identify a sex-specific pattern in the association between PA and HRQoL [21]; investigating the association between PA and HRQoL in the general adult population stratified by sex groups and PA intensity would be important and practical for health promotion programming and health policy change. Therefore, the current study aims to address this gap by investigating the association between PA and HRQoL and how this association differs across sex groups, various levels (low, moderate and high) and types of physical activity (leisure time and occupational) and different dimensions of HRQoL in a large population of Tehranian adults.

## Methods

### Study setting and participants

This cross-sectional study conducted using data from 6th phase (2014–2017) of the Tehran Lipid and Glucose Study (TLGS). To introduce the study briefly, it is a population-based cohort study starting in 1999 that focuses on determining the risk factors and prevention of non-communicable diseases. Participants of the TLGS were residence of district No.13 in Tehran. The TLGS has two main components including 1) cross-sectional prevalence study of non-communicable diseases (NCDs) and their associated risk factors (phase 1) and 2) ongoing prospective follow-up study in which data was collected every 3 years. Rational and design as well as study details have been published previously [22, 23]. For the current study, from all adult individuals who participated in the TLGS (*n* = 10,087) during 2014–2016 (the 6th phase), those with incomplete data on HRQoL (*n* = 1818) and physical activity (*n* = 469) were excluded; thus data from a total of 7800 adults were considered for the current analysis. The ethics committee of the Research Institute for Endocrine Sciences (RIES) of Shahid Beheshti University of Medical Sciences approved the study and all participants provided written informed consent.

### Measures

Trained interviewers collected information on demographic data including age, marital status, level of education and job status. Further information regarding smoking and chronic diseases including cancer, chronic kidney diseases, diabetes, hypertension and history of cardiovascular diseases were also collected. Body weight was measured using a digital scale while participants were in minimal clothes and without shoes. Height was measured while participants were without shoes in a standing position and their shoulders were in normal alignment. Body mass index (BMI) was calculated by dividing participant’s weight (in kilograms) by his/her height (in meters, squared) and then categorized into three groups including normal weight (BMI < 25 kg/m^2^), overweight (25 ≤ BMI < 30 kg/m^2^) and obese (BMI ≥ 30 kg/m^2^).

Information on physical activity was collected with the Iranian version of Modifiable Activity Questionnaire (MAQ) [24]. The psychometric properties of the Iranian version of the MAQ have been reported previously and the Iranian version of the questionnaire has been found to have high reliability and moderate validity [25]. For occupational activity, individuals were asked to report the number of hours per week they usually worked at a job and number of weekly hours they did house chores. In order to identify the minutes per week of occupational activity, the number of weekly hours of light, moderate and hard intensity activities were multiplied by 60 in each category over the past year. To calculate occupational activity, the number of minutes per week of each of the three categories of occupational activity was multiplied by the metabolic equivalent (MET) values (MET-min/wk) [26]. For leisure time activities, MET-min/wk. were calculated by multiplying the number of minutes per week of each activity by the MET. Total physical activity was expressed in MET-min/wk. as the sum of occupational and leisure time activities. Finally, low, moderate and high levels of physical activity were defined as values < 600 MET-min/wk., 600–3000 MET-min/wk. and ≥ 3000 MET-min/wk. respectively.

For assessment of HRQoL, participants completed the Short-Form 12-Item Health Survey version 2 (SF-12v2) which is a generic measure of perceived health status. This questionnaire encompasses 12 items and eight subscales (physical functioning, role physical, bodily pain, general health, vitality, social functioning, role emotional and mental health). The scores for each subscale ranged from 0 to 100, indicating the lowest and highest levels of health measured by the scale, respectively. Using the appropriate scoring algorithms, physical component summary (PCS) and mental component summary (MCS) scores were calculated. Previous findings confirmed validity and reliability of the Iranian version of SF-12v2 among the Iranian population [27].

### Statistical analysis

For normal and non-normal continuous variables mean ± sd and median (Q1-Q3) were reported respectively, while frequency and percentages were reported for categorical variables. Distribution of variables among groups was compared using independent samples T-test, one way ANOVA and Chi-square test. The HRQoL scores were compared among physical activity levels using analysis of covariance. The Spearman correlation coefficients were obtained to assess the relationship between physical activity and the HRQoL scores. For assessing the association between physical activity levels and poor HRQoL status, logistic regression analysis was performed. The poor HRQoL was defined as the first quartile of PCS or MCS and the odds ratios were estimated for physical activity groups by sex. All models were adjusted for the variables which were significantly different among physical activity levels. Statistical analysis was performed using SPSS package, version 22. *P* values of less than 0.05 were considered to be statistically significant.

## Results

Mean age of participants was 46.4 ± 14.9 years and 45.6% of them were male. Table 1 indicates descriptive statistics of study participants by sex groups. As it is shown, the majority of participants were married (76.3%). More women had a primary level of education or less compared to men (27.5 vs. 19.5% respectively) and more men had academic degrees compared to women (38.3 vs. 33.0% respectively). Most of the women were housewives (70.8%) and most of the men were employed (73.7%). There were significant differences in distribution of men and women across different levels of physical activities (*p* < 0.001). The percentage of men with a high level of physical activity was twice as high as women (23.7 vs. 12.1% respectively). A significantly higher percentage of men were smokers compared to women (24.9 vs. 3.9% respectively). Less than one third of men (29.2%) and women (27.9%) had normal weight. In terms of chronic diseases, there were no significant differences in distribution of diabetes and cancer between men and women. On the other hand, a higher percentage of men had hypertension and a history of CVD compared to women; while a higher percentage of women suffered from chronic kidney diseases.

**Table 1:** Descriptive statistics of study participants

	Total (*n* = 7800)	Men (*n* = 3557)	Women (*n* = 4243)	*P* value
Age (year)	46.4 ± 14.9	46.6 ± 15.8	46.2 ± 14.3	0.313
Marital status n(%)				
Married	5950 (76.3)	2770 (77.9)	3180 (75.1)	0.003
Unmarried (single/widowed/divorced)	1841 (23.6)	785 (22.1)	1056 (24.9)	
Level of education n(%)				
Illiterate/Primary	1860 (23.9)	695 (19.5)	1165 (27.5)	< 0.001
Secondary	3175 (40.7)	1500 (42.2)	1675 (39.5)	
Higher	2760 (35.4)	1360 (38.3)	1400 (33.0)	
Job status n(%)				
Unemployed/student/housewife	3275 (42.0)	272 (7.6)	3003 (70.8)	< 0.001
Unemployed, but had other sources of income	1079 (13.8)	664 (18.7)	415 (9.8)	
Employed	3442 (44.2)	2620 (73.7)	822 (19.4)	
Physical activity n(%)				
Low	2552 (23.7)	1368 (38.5)	1184 (27.9)	< 0.001
Moderate	3892 (49.9)	1347 (37.9)	2545 (60.0)	
High	1356 (17.4)	842 (23.7)	514 (12.1)	
Leisure time METS^*^	9.3 (4.1–19)	11.0 (4.8–22.3)	7.9 (4.0–16.5)	< 0.001
Occupational METS^*^	11.1 (2.8–27.8)	6.9 (0–35.7)	13.9 (5.6–27.8)	< 0.001
Total METS^*^	18.1 (7.0–37.4)	17.1 (4.9–47.6)	18.9 (8.3–33.3)	0.172
Smoking n(%)				
Yes	1049 (13.5)	884 (24.9)	165 (3.9)	< 0.001
No	6750 (86.5)	2673 (75.1)	4077 (96.1)	
Body weight status n(%)				
Normal weight	2210 (28.5)	1036 (29.2)	1174 (27.9)	< 0.001
Overweight	3240 (41.7)	1650 (46.5)	1590 (37.7)	
Obese	2312 (29.8)	863 (24.3)	1449 (34.4)	
Diabetes n(%)				
Yes	1058 (15.1)	474 (14.7)	584 (15.4)	0.425
No	5942 (84.9)	2741 (85.3)	3201 (84.6)	
Hypertension n(%)				
Yes	1721 (22.1)	841 (23.7)	880 (20.8)	0.003
NO	6061 (77.9)	2714 (76.3)	3347 (79.2)	
History of CVD/CHD n(%)				
Yes	639 (8.2)	368 (10.3)	271 (6.4)	< 0.001
No	7161 (91.8)	3189 (89.7)	3972 (93.6)	
Chronic kidney diseases n(%)				
Yes	1762 (23.0)	536 (15.4)	1226 (29.4)	< 0.001
No	5891 (77.0)	2947 (84.6)	2944 (70.6)	
Cancer n(%)				
Yes	99 (1.3)	38 (1.1)	61 (1.4)	0.147
NO	7701 (98.7)	3519 (98.9)	4182 (98.6)	

^*^Median and interquartile range (Q1-Q3)

Table 2 indicates the distribution of socio-demographic characteristics and chronic diseases across different levels of physical activity in men and women. Except for level of education in men and cancer in both sexes, there were significant differences in distribution of remaining socio-demographic factors and chronic diseases in men and women with low, moderate and high levels of physical activity. Therefore, all regression models were adjusted for those variables which were significantly different among physical activity levels.

**Table 2:** Socio-demographic characteristics and distribution of body weight and health status of study participants across different groups of physical activity

	Men			*P* value	Women			*P* value
	Low (*n* = 1368)	Moderate (*n* = 1347)	High (*n* = 842)		Low (*n* = 1184)	Moderate (*n* = 2545)	High (*n* = 514)	
Age (year)	47.1 ± 14.9	47.8 ± 16.7	43.6 ± 15.0	< 0.001	47.5 ± 15.9	46.2 ± 13.6	43.4 ± 12.7	< 0.001
Marital status n(%)								
Married	1113 (81.5)	1040 (77.2)	617 (73.3)	< 0.001	805 (68.2)	1985 (78.1)	390 (76.0)	< 0.001
Unmarried (single/widowed/divorced)	253 (18.5)	307 (22.8)	225 (26.7)		375 (31.8)	558 (21.9)	123 (24.0)	
Level of education n(%)								
Illiterate/Primary	263 (19.3)	261 (19.4)	171 (20.3)	0.054	394 (33.3)	671 (26.4)	100 (19.5)	< 0.001
Secondary	549 (40.2)	568 (42.2)	383 (45.5)		418 (35.3)	1045 (41.1)	212 (41.3)	
Higher	554 (40.6)	518 (38.5)	288 (34.2)		372 (31.4)	827 (32.5)	201 (39.2)	
Job status n(%)								
Unemployed/student/housewife	105 (7.7)	116 (8.6)	51 (6.1)	< 0.001	836 (70.6)	1869 (73.5)	298 (58.1)	< 0.001
Unemployed, but had other sources of income	231 (16.9)	333 (24.7)	100 (11.9)		132 (11.1)	243 (9.6)	40 (7.8)	
Employed	1031 (75.4)	898 (66.7)	691 (82.1)		216 (18.2)	431 (16.9)	175 (34.1)	
Body weight status n(%)								
Normal weight	363 (26.6)	384 (28.6)	289 (34.4)	< 0.001	335 (28.6)	692 (27.4)	147 (28.7)	< 0.001
Overweight	616 (45.2)	667 (49.6)	367 (43.7)		399 (34.0)	962 (38.1)	229 (44.6)	
Obese	385 (28.2)	294 (21.9)	184 (21.9)		438 (37.4)	874 (34.6)	137 (26.7)	
Diabetes								
Yes	209 (16.8)	188 (15.6)	77 (10.1)	< 0.001	199 (18.9)	335 (14.8)	50 (10.8)	< 0.001
Hypertension								
Yes	341 (25.0)	349 (25.9)	151 (17.9)	< 0.001	310 (26.4)	500 (19.7)	70 (13.7)	< 0.001
History of CVD/CHD								
Yes	159 (11.6)	166 (12.3)	43 (5.1)	< 0.001	104 (8.8)	149 (5.9)	18 (3.5)	< 0.001
Chronic kidney diseases n(%)								
Yes	201 (15.0)	238 (18.0)	97 (11.8)	< 0.001	376 (32.4)	745 (29.8)	105 (20.7)	< 0.001
Cancer n(%)								
Yes	10 (0.7)	18 (1.3)	10 (1.2)	0.286	22 (1.9)	33 (1.3)	6 (1.2)	0.350

Comparison of HRQoL scores among different levels of physical activity by sex are indicated in Table 3. The HRQoL scores were significantly different among men with various levels of physical activity in all subscales except for role physical, bodily pain and social functioning subscales (*p* < 0.05). In women, HRQoL scores were significantly different among those with various levels of physical activity in all subscales except for social functioning, role emotional and mental health subscales (*p* < 0.01). In terms of HRQoL physical and mental summary scores, PCS in both men and women and MCS only in men were significantly different among different levels of physical activity.

**Table 3:** Mean health-related quality of life scores among men and women with different levels of physical activity

	Men			*P* value	Women			*P* value
	Low	Moderate	High		Low	Moderate	High	
SF-12								
Physical Function	82.3 ± 1.0	85.9 ± 1.0	86.0 ± 1.1	**< 0.001**	72.4 ± 1.6	78.3 ± 1.5	80.9 ± 1.8	**< 0.001**
Role Physical	81.7 ± 0.9	82.3 ± 1.0	82.3 ± 1.1	0.715	66.8 ± 1.4	70.9 ± 1.4	73.3 ± 1.7	**< 0.001**
Bodily pain	83.0 ± 1.1	83.5 ± 1.0	82.8 ± 1.2	0.726	70.5 ± 1.5	73.2 ± 1.5	74.9 ± 1.8	**0.002**
General Health	42.2 ± 1.1	45.7 ± 1.1	45.3 ± 1.2	**< 0.001**	38.3 ± 1.3	40.1 ± 1.2	42.1 ± 1.5	**0.004**
PCS	48.1 ± 0.4	49.3 ± 0.4	48.7 ± 0.4	**< 0.001**	45.0 ± 0.5	46.8 ± 0.5	47.8 ± 0.6	**< 0.001**
Vitality	62.7 ± 1.2	65.3 ± 1.2	68.5 ± 1.4	**< 0.001**	51.3 ± 1.6	56.9 ± 1.5	61.7 ± 1.8	**< 0.001**
Social Function	82.1 ± 1.2	81.8 ± 1.2	82.3 ± 1.4	0.875	69.7 ± 17	70.7 ± 1.6	72.0 ± 1.9	0.309
Role Emotional	75.5 ± 1.1	75.2 ± 1.0	77.7 ± 1.2	**0.024**	64.3 ± 1.5	65.9 ± 1.4	66.4 ± 1.7	0.133
Mental Health	71.8 ± 1.1	70.7 ± 1.0	73.8 ± 1.2	**0.007**	58.3 ± 1.4	59.2 ± 1.3	60.1 ± 1.6	0.356
MCS	48.8 ± 0.5	48.3 ± 0.5	49.9 ± 0.6	**0.003**	42.9 ± 0.7	43.3 ± 0.7	43.8 ± 0.8	0.337

The significance level has been considered *p* < 0.05; hence as all bold numbers in the table are less than 0.05, all of them are significant" to "*p* < 0.05 are in bold

The correlations between physical activity in both occupational and leisure time levels and HRQoL scores are reported in Table 4. In men, leisure time physical activities were significantly correlated to all HRQoL subscales except for bodily pain, social functioning and role emotional. In addition, occupational physical activities were significantly correlated only to vitality. In women, leisure time physical activities were significantly correlated to all HRQoL subscales except for bodily pain. Furthermore, occupational physical activities were significantly correlated to physical functioning, role physical, general health and vitality.

**Table 4:** Association between physical activity and health-related quality of life by sex

	Men			Women		
	Total	Leisure time	Job	Total	Leisure time	Job
SF-12						
Physical Function	0.11^**^	0.10^**^	0.03	0.14^**^	0.05^*^	0.12^**^
Role Physical	0.04^*^	0.05^*^	0.0	0.11^**^	0.06^**^	0.09^**^
Bodily pain	0.01	0.02	−0.01	0.06^**^	0.04	0.03
General Health	0.10^**^	0.14^**^	−0.01	0.10^**^	0.05^*^	0.05^**^
PCS	0.08^**^	0.10^**^	0.0	0.13^**^	0.05^*^	0.10^**^
Vitality	0.12^**^	0.14^**^	0.05^**^	0.15^**^	0.12^**^	0.10^**^
Social Function	0.0	0.02	0.0	0.04^*^	0.05^*^	0.02
Role Emotional	0.01	0.01	0.02	0.04^**^	0.07^**^	0.02
Mental Health	0.03	0.05^*^	0.01	0.05^**^	0.06^**^	0.02
MCS	0.03	0.04	0.02	0.04^**^	0.08^**^	0.01

Spearman correlation coefficients have been reported

^*^p < 0.05, ^**^p < 0.01

The significance level has been considered *p* < 0.05; hence as all bold numbers in the table are less than 0.05, all of them are significant" to "*p* < 0.05 are in bold

Table 5 indicates the odds ratios of reporting poor physical and mental HRQoL for different levels of physical activity in men and women. In men, the chance of reporting poor PCS was significantly higher in those with low levels of physical activity compared to those with high levels of physical activity, only in the unadjusted model (OR: 1.63, 95%CI: 1.28–2.09; *p* < 0.001). In addition, men with both low and moderate levels of physical activity had significantly a higher chance of reporting poor MCS in both unadjusted (OR: 1.32, 95%CI: 1.03–1.69; *p* = 0.028 and OR: 1.35, 95%CI: 1.06–1.73; *p* = 0.017 respectively) and adjusted models (OR: 1.46, 95%CI: 1.11–1.91; *p* = 0.007 and OR: 1.55, 95%CI: 1.18–2.04; *p* = 0.002 respectively). In the unadjusted model for women, the chances of reporting poor PCS were significantly higher in those with low and moderate levels of physical activity (OR:2.65, 95% CI:1.96–3.59; *p* < 0.001 and OR:1.60, 95% CI:1.20–2.11; *p* = 0.001 respectively) compared to their counterparts with high level of physical activity. After adjusting for confounding variables, only women with low level of physical activity had a significantly higher chance of reporting poor PCS (OR:2.39, 95% CI:1.63–3.49; *p* < 0.001) compared to those with high levels of physical activity. Furthermore, the chance of reporting poor MCS was significantly higher in women with low levels of physical activity compared to those with high levels of physical activity, only in unadjusted model (OR: 1.38, 95%CI: 1.03–1.84; *p* = 0.029).

**Table 5:** Odds ratios and 95% confidence intervals for poor health-related quality of life among men and women

	Level of physical activity	PCS	*P* value	MCS	*P* value
		OR (95%CI)		OR (95%CI)	
Men					
Unadjusted Model	-High	1		1	
	-Moderate	1.01 (0.79–1.28)	0.96	1.35 (1.06–1.73)	**0.017**
	-Low	1.63 (1.28–2.09)	**< 0.001**	1.32 (1.03–1.69)	**0.028**
Adjusted Model	-High	1		1	
	-Moderate	0.76 (0.57–1.02)	0.066	1.55 (1.18–2.04)	**0.002**
	-Low	1.27 (0.95–1.69)	0.103	1.46 (1.11–1.91)	**0.007**
Women					
Unadjusted Model	-High	1		1	
	-Moderate	1.60 (1.20–2.11)	**0.001**	1.28 (0.98–1.67)	0.067
	-Low	2.65 (1.96–3.59)	**< 0.001**	1.38 (1.03–1.84)	**0.029**
Adjusted Model	-High	1		1	
	-Moderate	1.32 (0.93–1.86)	0.119	1.18 (0.89–1.58)	0.255
	-Low	2.39 (1.63–3.49)	**< 0.001**	1.26 (0.92–1.74)	0.147

Model 1 is unadjusted model and model 2 is adjusted for age, marital status, level of education, job status, body weight status, history of diabetes, CVD, hypertension and chronic kidney diseases

The significance level has been considered *p* < 0.05; hence as all bold numbers in the table are less than 0.05, all of them are significant" to "*p* < 0.05 are in bold

## Discussion

This study aims to explore the association between PA and HRQoL and further indicate how this association varies across sex groups, various levels (low, moderate and high) and types of PA (leisure time and occupational) and different dimensions of HRQoL in Tehranian adults. The findings of the current study showed that individuals with higher levels of PA reported better HRQoL in different domains. Our findings replicate results of other studies conducted in Iran regarding positive association between PA and HRQoL specifically in different groups of women [18, 19]. Similarly, several cross-sectional [5, 28, 29] and longitudinal studies [14, 16] conducted in different countries indicated better HRQoL in more active individuals.

The current study found that leisure-time PA was significantly correlated to all HRQoL subscales except bodily pain in both men and women, and except social functioning and role emotional in just men. This finding implies that men benefit from leisure-time PA levels similar to women in their physical HRQoL, but do not have the same mental HRQoL benefits that women do. Our findings are in agreement with previous studies indicating significant cross-sectional and longitudinal associations between leisure physical activity and domains of HRQoL [13–16]; however, there were some differences in this association by gender and domain of HRQoL. While some studies showed no sex difference in the effects of PA on HRQoL [15], several studies indicated a sex-specific pattern [13, 14, 16] with more mental HRQoL benefits in women compared to men [13, 14], consistent with our findings.

Current findings indicate that occupational physical activities were positively and significantly correlated only to vitality in men and to physical functioning, role physical, general health and vitality in women. Few studies investigated association between occupational PA and HRQoL. One study found that work related activities have positive associations with physical functioning and bodily pain in female students and negative associations with physical functioning and bodily pain in male students [15]. Another study by Paivarinne et al. identified a negative association between occupational PA and physical HRQoL in young adult men [17]; however, Jurakic et al. reported positive association between occupational PA and role physical in men [13]. In contrast, Kaleta and colleagues found no significant effect of occupational physical activity on the shaping of self-perceived heath status in men or women [30]. The observed differences in association between occupational PA and HRQoL by sex may be explained by the types of jobs that men and women have. While many studies have explored the significance of leisure-time physical activity in relation to HRQoL; there is a paucity of research related to the association of occupational physical activity and HRQoL. This gap in evidence suggests the need to further explore the association between occupational PA and HRQoL in men and women.

Finally, in terms of poor physical and mental HRQoL, our study found that physical domain in both men and women and mental domain in men were significantly different among different levels of physical activity. Our study suggested that after adjusting for confounders, the chance of reporting poor MCS was significantly higher in men with both low and moderate levels of physical activity compared to those with high level of physical activity. On the other hand, women with low levels of physical activity had significantly poorer PCS in comparison to those with higher levels of activity. To further elaborate, women experience greater benefits to their physical HRQoL, while men experience greater benefits to their mental HRQoL with higher levels of physical activity. One reason for this discrepancy may be due to gender differences in biological structure and function in men and women. Existing evidence indicate that weight and height at birth, vital capacity, muscle mass, cardiovascular physiology and brain function differ in men and women; these differences may contribute to health benefits derived from physical activity [31]. In addition, health advantages of exercising vary in men and women based on the level, mode and intensity of the PA they participate in [31]. Another reason for this sex difference may be due to differing motives for exercising. Craft et al. indicated that reasons for exercise better predicted HRQoL compared to exercise itself. Reasons for exercising such as toning, achieving improved fitness and weight reduction was more common among women, while having fun and deriving pleasure was the main reason for exercising among men [32]. The difference in the type of exercise as well as the motivation to exercise could provide an explanation for the varying physical and mental HRQoL patterns in men and women.

Our findings for the first time present the sex-specific associations between PA and HRQoL considering type and intensity of PA in an urban population of Iran. The current study had some limitations. Due to the cross-sectional design, ascertaining a causal relationship between HRQoL and PA levels was not possible. Second, the results were derived from self-reported variables subject to self-report bias. Additionally, previous reports found moderate validity for the Iranian version of the MAQ instrument; therefore, the findings should be interpreted with caution. Lastly, our study sample consisted of adults residing in Tehran thus potentially limiting the generalizability of the findings to a wider population.

## Conclusion

The current study showed the significant benefits to HRQoL experienced with greater PA in both men and women. This positive association was mainly observed in women’s physical HRQoL and men’s mental HRQoL. These sex-specific findings could be considered to motivate participation in PA programs in the future health promotion interventions in urban populations in Iran and other similar communities.

## Data Availability

Data would be available on the request of corresponding author based on TLGS rules.

## Abbreviations

HRQoL: Health-related quality of life
MAQ: Modifiable Activity Questionnaire
MCS: Mental component summary
MET: Metabolic equivalent
OR: Odds ratio
PA: Physical activity
PCS: Physical component summary
RIES: Research Institute for Endocrine Sciences
SF-12v2: Short-Form 12-Item Health Survey version 2
TLGS: Tehran Lipid and Glucose Study
